# *OsWRKY93* Dually Functions Between Leaf Senescence and in Response to Biotic Stress in Rice

**DOI:** 10.3389/fpls.2021.643011

**Published:** 2021-03-22

**Authors:** Yanyun Li, Shuting Liao, Pengying Mei, Yueyun Pan, Yu Zhang, Xiangzi Zheng, Yakun Xie, Ying Miao

**Affiliations:** ^1^Fujian Provincial Key Laboratory of Plant Functional Biology, Fujian Agriculture and Forestry University, Fuzhou, China; ^2^College of Life Sciences, Fujian Agriculture and Forestry University, Fuzhou, China

**Keywords:** *OsWRKY93*, rice, flag leaf, senescence, biotic stress

## Abstract

Cross talking between natural senescence and cell death in response to pathogen attack is an interesting topic; however, its action mechanism is kept open. In this study, 33 *OsWRKY* genes were obtained by screening with leaf aging procedure through RNA-seq dataset, and 11 of them were confirmed a significant altered expression level in the flag leaves during aging by using the reverse transcript quantitative PCR (RT-qPCR). Among them, the *OsWRKY2*, *OsWRKY14*, *OsWRKY26*, *OsWRKY69*, and *OsWRKY93* members exhibited short-term alteration in transcriptional levels in response to *Magnaporthe grisea* infection. The CRISPR/Cas9-edited mutants of five genes were developed and confirmed, and a significant sensitivity to *M. oryzae* infection was observed in *CRISPR OsWRKY93-edited* lines; on the other hand, a significant resistance to *M. oryzae* infection was shown in the enhanced expression *OsWRKY93* plants compared to mock plants; however, enhanced expression of other four genes have no significant affection. Interestingly, ROS accumulation was also increased in *OsWRKY93* enhanced plants after flg22 treatment, compared with the controls, suggesting that *OsWRKY93* is involved in PAMP-triggered immune response in rice. It indicated that *OsWRKY93* was involved in both flag leaf senescence and in response to fungi attack.

## Introduction

Rice is the main food crop of the developing world. However, the increase of yield is seriously restricted by flag leaf senescence in rice. The flag leaf, the uppermost leaf in the rice plant, is thought to contribute highly to what is accumulated in grain (Ghosh et al., 1990; Li et al., 1998). Delaying the senescence of rice leaves and prolonging the photosynthesis time are beneficial for increasing the rice yield, and the yield can increase by about 2% after flag leaf senescence is delayed for 1 day (Ma and Lu, 1990). Therefore, studying the mechanism of flag leaf senescence is essential to improving the yield of rice grain.

Leaf senescence is the final stage of leaf development. As an organ level senescence, leaf senescence is a crucial means for plants to reallocate nutrients and valuable substances from senescent leaves to reproducing seeds, eventually maximizing reproductive success (Himelblau and Amasino, 2001). Leaf senescence is a strictly organized process finely governed by developmental age. However, leaf senescence is also influenced by various internal and environmental signals that are integrated with age information (Lim et al., 2007). The internal factors that affect leaf senescence include developmental cues and reproductive development as well as phytohormones (Gan and Amasino, 1995; Pic et al., 2002; Riefler et al., 2006). The environmental cues include various stresses such as extreme temperatures, nutrient deficiency, drought, radiation, and infection from pathogens. Interestingly, the leaf transcriptome varies immensely accompanying the onset and progression of leaf senescence. It was previously reported that 20 different families of transcription factors that are transcriptionally up-regulated in senescent leaves remarkably contain several large groups such as NAC, WRKY, C2H2-type zinc finger, AP2/EREBP, and MYB proteins (Guo and Gan, 2005).

Among these large groups, WRKY proteins are plant specific transcription factors that are especially believed to play central roles in regulating senescence. All WRKY proteins contain at least one WRKY domain that is composed of a zinc finger structure and a 60-amino acid region with WRKYGQK at the N-terminal end. The WRKY domain is a DNA-binding domain that binds directly to various W-box variants (Eulgem et al., 2000; Yu et al., 2001). To date, many WRKY TFs regulating leaf senescence have been characterized in *Arabidopsis*. *WRKY6* is highly induced during leaf senescence (Robatzek and Somssich, 2001). *WRKY45* positively regulates age-triggered leaf senescence through interacting with a DELLA protein, RGL1 (Chen L. et al., 2017). Another well-known WRKY member, *WRKY53* plays a regulatory role in the early events of leaf senescence (Hinderhofer and Zentgraf, 2001; Miao et al., 2004). Overexpression of *WRKY75* accelerates age-dependent leaf senescence (Guo et al., 2017). In rice, WRKY family has over 102 members (Xie et al., 2005). However, relatively few *OsWRKY* members involved in leaf senescence have been examined. For instance, overexpressing *OsWRKY5* promotes leaf senescence under natural and dark-induced senescence conditions (Kim et al., 2019). Heterologous expression of *OsWRKY23* promotes dark-induced leaf senescence in Arabidopsis (Jing et al., 2009). *OsWRKY42* enhances leaf senescence by repressing the expression of *OsMT1d* to induce reactive oxygen species (ROS) in rice (Han et al., 2014).

The WRKY family is also known for being the key player in plant biotic stress response. The initial study investigated the expression of WRKY TFs in rice response to *M. oryzae* and found that 15 *OsWRKYs* were induced upon pathogen infection (Ryu et al., 2006). Subsequent research revealed more details about the involvement of many *OsWRKYs* in plant defense. At least nine *OsWRKYs* have been identified to regulate rice response to *M. oryzae* positively. For example, overexpression of *OsWRKY31*, *OsWRKY45*, *OsWRKY47*, *OsWRKY53*, or *OsWRKY67* in rice plants enhances resistance to *M. oryzae* (Chujo et al., 2007; Shimono et al., 2007; Zhang et al., 2008; Wei et al., 2013; Vo et al., 2018). On the contrary, several *OsWRKY* members function as negative regulators of the rice response to *M. oryzae* infection. For instance, through suppressing JA signaling-related genes, *OsWRKY42* negatively regulate rice response to *M. oryzae* (Cheng et al., 2015). Overexpression of *OsWRKY28* or *OsWRKY76* in rice plants resulted in increased susceptibility to *M. oryzae* (Chujo et al., 2013; Yokotani et al., 2013).

In this study, the transcriptome analysis shows that 33 *OsWRKY* members in rice flag leaves are differentially expressed during plant aging. Besides, RT-qPCR analysis displayed that the expression of five *OsWRKY* genes were altered in Guy11-treated rice plants. The Crispr/Cas9-edited mutants of five *OsWRKY* genes were developed and confirmed. Genetic analysis reveals that enhanced expression of *OsWRKY93* resulted in an enhanced resistance to *M. oryzae* infection in rice. This finding suggests that *OsWRKY93* plays a role in the defense response and is also associated with the regulation of flag leaf senescence in rice. All in all, this study provides a new candidate gene for in depth understanding of the regulatory mechanisms of pathogen induced leaf senescence, helping in breeding high yield and disease resistant crops.

## Materials and Methods

### Plant Materials and Growth Conditions

The rice (*Oryza sativa* L. subsp. *japonica*) of the Kitaake accession was used for generating *OsWRKY2, OsWRKY14, OsWRKY26, OsWRKY69*, and *OsWRKY93* transgenic plants with increased *OsWRKY2, OsWRKY14, OsWRKY26, OsWRKY69*, and *OsWRKY93* expression level via a transcriptional activator containing four copies of VP16 (i.e., VP64), and named *OsWRKY_*VP*__64_* (Sadowski et al., 1988; Yaghmai and Cutting, 2002). Rice plants were grown in the growth chamber at 30°C for 12 h (day) and 20°C for 12 h (night) or under outdoor conditions (natural long-day conditions) in Fuzhou Fujian Province, China, from April to September.

### Identification of CRISPR/Cas9-Edited Mutants

The *OsWRKY2*, *OsWRKY14*, *OsWRKY26*, *OsWRKY69*, and *OsWRKY93* CRISPR transgenic plants were produced by the Biogle company (Hangzhou, China). Genomic DNA from individual transgenic plants was isolated using Edwards buffer (Edwards et al., 1991) for PCR analysis. The PCR products were amplified with *OsWRKY93*-specific primers and were sequenced directly. The *OsWRKY93*-specific primers were designed for amplifying targeted regions of *OsWRKY93* (Supplementary Table S2).

### Pathogen Inoculation

*M. oryzae* strain Guy11 was used in this study. At the three-leaf stage, rice seedlings were spray-inoculated with the spore suspension of *M. oryzae* (1 × 10^5^ spores/ml in water containing 0.02% Tween 20). Subsequently, the inoculated plants were incubated in the dark at high humidity for 24 h and transferred to a growth chamber at 24°C with 12 h of light and 12 h of darkness. The disease lesions in the infected leaves were observed, and were scanned at 0, 1, 3, 4 days post-inoculation (dpi).

### Darkness Treatment

Kitaake, NIP, *oswrky93-1* mutant and the T2 generation OsWRKY93_*v*__*p*__64_ plants were cultured in soil for 39 days after germination. The fully expanded part of the sixth leaves were cut into 1–2 cm pieces and pooled, and then the leaf pieces were suspended in 3mM MES (pH5.8) buffer and cultured in the dark at 28°C for 0, 24, 36, 48, 60, 72, 84, and 96 h. The color changes of leaves were observed and photographed. Three biological replicates were used.

### Chlorophyll Measurements

The chlorophyll content of flag leaves were measured using a chlorophyll meter (DUALEX SCIENTIFIC). For measurement 3–4 points in the central region of the leaf were picked up.

### Reverse Transcription Quantitative PCR

Three-leaf stage rice seedlings were spray-inoculated with Guy11 (1 × 10^5^ spores/ml) and water, and leaf samples were collected at 0, 24, 48, 72, 96, and 108 hrs post-inoculation (hpi). Two biological replicates were tested, and each biological replicate contains leaves from three independent plants. Total RNA was extracted from those leaf samples using TRIzol reagent (Invitrogen), followed by cDNA synthesis with RevertAid Reverse Transcriptase (Thermo Fisher Scientific). Quantitative PCR was performed using TransStart Green qPCR SuperMix Kit (TransGen Biotech, China) and the indicated primers (Supplementary Table S1). The rice actin1 (*OsACTIN1*) gene was selected as an internal control.

### ROS Assay

Oxidative bursts were measured using a luminal-based assay with leaf discs from 5-week-old plants. The leaf discs were incubated in sterile water overnight, and then water was replaced with 20 μM luminal and 2.5 μg/ml peroxidase. To measure ROS, leaf discs were treated with 1 μM flg22 or water (Ctrl). Immediately, the luminescence was measured at 3 min intervals with a Varioskan LUX Multimode Microplate Reader (Thermo Fisher Scientific). Then 3–5 replications were carried out for each sample.

## Results

### Expression Patterns of *OsWRKYs* in Rice Flag Leaves During Natural Senescence

**FIGURE 1:**
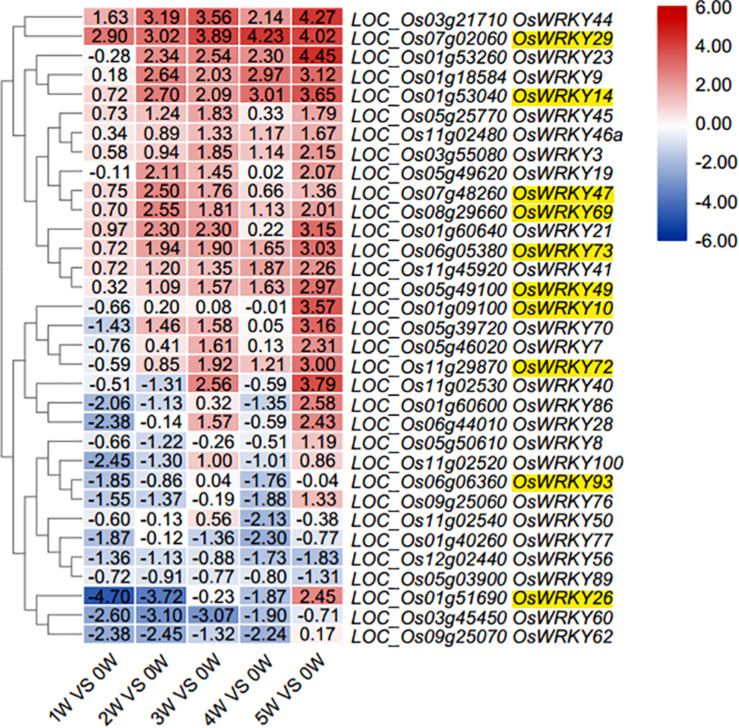
Heat map diagram of relative gene expression levels of 33 *OsWKRYs* from total 102 *WRKYs* (Supplementary Dataset S1) in rice flag leaves at six stages during aging. Developmental stages comprising six stages of flag leaf (0, 1, 2, 3, 4, and 5 weeks after heading). Expression values were scaled by Log2Fold change ≥ 1 and FDR < 0.05 normalized to 0W stage of flag leaf development. 10 *OsWRKY* candidates are indicated with yellow highlight.

To monitor the transcriptional changes in rice flag leaves during natural senescence, a genome-wide transcriptome analysis was carried out in flag leaf tissue of the *Nipponbare* through massive RNA sequencing. For generation of RNA-seq libraries, six flag leaf samples were taken. The first sample of the flag leaf was collected at the heading stage when the flag leaf was fully expanded [0 weeks after heading (WAH) and named 0W]; chlorophyll content is higher in 1w than 0w, and then it is gradually decreased from 1w to 5w; the following five flag leaf samples were collected every week (named 1W, 2W, 3W, 4W, and 5W, respectively, 0W used as control). The onset of leaf senescence coincides with the start of Chlorophyll (Chl) degradation, while the initiation of leaf senescence is before Chl degradation. Therefore, the senescence initiation of flag leaves started at the time period between 0W and 2W (Supplementary Figure S1). Through RNA-Seq analysis, the expression patterns of 102 *OsWRKY* family members in rice flag leaves during aging stages were investigated (Supplementary Dataset S1). EdgeR program was used for differential expression analysis of *OsWRKY* genes between any of the six samples (Nikolayeva and Robinson, 2014). In comparison with the control (0W), a differential expression profile of a total thirty-three *OsWRKY* genes were exhibited during natural senescence of flag leaves (Figure 1 and Supplementary Dataset S1).

**FIGURE 2:**
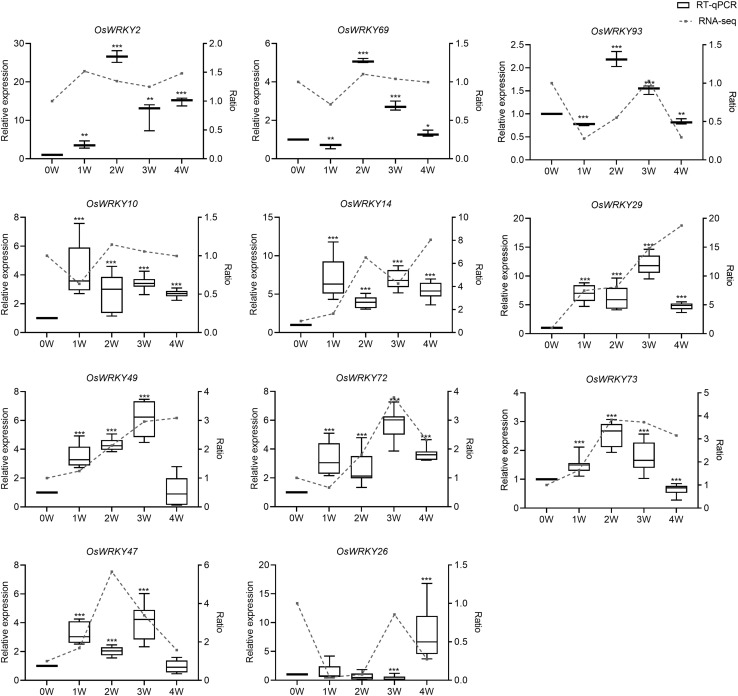
Analyses of several *OsWRKYs* expression level in rice flag leaves during natural senescence. The expression level was assessed by RT-qPCR. All values were normalized to *OsACTIN* expression. Box-and-whisker plots show the median value (horizontal lines), interquartile range (boxes), and minimum and maximum values (whiskers). Three biological replicates and three technique replicates were used. The broken-line graphs indicate expression profiles of 11 *OsWRKYs* from RNA-seq dataset. Asterisks indicate significant differences relative to the 0W controls calculated using the Student *t*-test: *P < 0.05; **P < 0.01; and ***P < 0.001. The leaf *Y*_axis denotes relative expression by RT-qPCR. The right *y*-axis denotes ratio of the fold change of RPKM compared with 0W by RNA-seq. 0W means 0 week after heading.

To further confirm the differential expression of thirty-three *OsWRKY* genes during natural senescence according to transcriptome data (Figure 2 and Supplementary Dataset S1), all of 33 *OsWRKY* genes were checked by RT-qPCR, the transcript levels of eight OsWRKYs (*OsWRKY2*, *OsWRKY10*, *OsWRKY14*, *OsWRKY29, OsWRKY47*, *OsWRKY49*, *OsWRKY72*, and *OsWRKY73*) were immediately up-regulated in 1W-vs-0W comparison, while that of three OsWRKYs (*OsWRKY69*, *OsWRKY93*, *OsWRKY26*) were slightly down-regulated in 1W-vs-0W comparison then up-regulated in 2W vs. 0W again (Figure 2), suggesting that they are senescence-related *OsWRKY* genes. Among the 11 *OsWRKY* genes, *OsWRKY2*, *OsWRKY69*, and *OsWRKY93* shared a similar expression pattern in rice flag leaves that the transcript level increased and peaked at the second week after heading (2W) and declined afterward compared with the 0W control. The expression of *OsWRKY10* and *OsWRKY14* reached the highest level at 1W and remained relatively high afterward. The level of *OsWRKY26* mRNA was slightly increased at 1W and then stayed low level at 2W and 3W and suddenly highly increased at 4W. At 3 weeks after heading, the expression of *OsWRKY29*, *OsWRKY47*, *OsWRKY49*, and *OsWRKY72* was significantly higher than other controls and began to decrease later (Figure 2). Overall, the results of RT-qPCR were similarly consistent with the RNA-seq data except *OsWRKY26* and *OsWRKY47* (Figure 2 broken line).

### Expression Profiles of *OsWRKYs* in Response to Pathogen Infection

**FIGURE 3:**
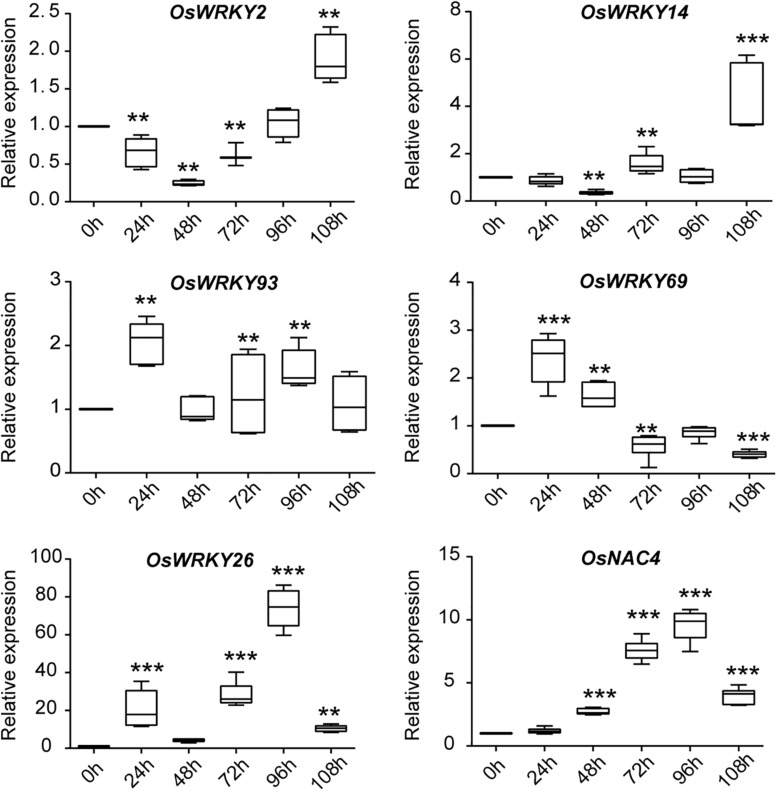
Expression analysis of five *OsWRKY* genes and the defense-related marker gene Os*NAC4* in response to *M. oryzae* infection. qRT-PCR analysis of five *OsWRKYs* and *OsNAC4* in WT at 0, 24, 48, 72, 96, and 108 h after pathogen treatment. The *Y*-axis represents the relative expression level normalized to *OsACTIN*. Box-and-whisker plots show median value (line within box), interquartile range (boxes), and minimum and maximum values (whiskers). Three biological replicates and three technique replicates were used. Asterisk indicate significant differences (**P < 0.01, and ***P < 0.001) based on Student *t*-test compared to 0 h.

In nature, plants are often attacked by various pathogens, leading to senescence and even death of plants. In this case, plants will initiate a series of immune defense responses to fight back. A number of WRKY family TFs are involved in regulation of both leaf senescence and pathogen defense response, evidently through the ROS and SA pathways, both of which play an important role in leaf senescence and defense responses induced by pathogens (Zhang et al., 2020). To investigate whether these 11 *OsWRKYs* are induced by infection from pathogens, we performed RT-qPCR (Figure 3). For pathogen treatment, three-leaf-stage rice seedlings were spray-inoculated with *Magnaporthe oryzae* strain Guy11. The infected leaf samples were collected every 24 h for near 5 days. The defense-related gene, *OsNAC4*, was used as a positive marker control, showing increased transcript levels in the infected leaves (Kaneda et al., 2009). Among 11 *OsWRKYs, OsWRKY2*, *OsWRKY14*, *OsWRKY26*, *OsWRKY69*, and *OsWRKY93* were induced by *M. oryzae* infection. For instance, *OsWRKY69* and *OsWRKY93* had slightly elevated mRNA levels in infected plants, and they were exclusively expressed at the early stage of infection. On the contrary, *OsWRKY2* and *OsWRKY14* were up-expressed at the late stage after infection. Specifically, the expression of *OsWRKY26* was strongly up-regulated at 96 h after inoculation with Guy11. Taken together, the five *OsWRKYs* appear to play roles in *M. oryzae* mediated resistance.

We summarized the expression profiles of five *OsWRKYs* genes both after pathogen infection and during plant aging and showed that *OsWRKY2* was down-regulated, which might mean no resistance and no senescence; *OsWRKY14* was down-regulated after infection but up-regulated during plant aging, which might imply senescence but no resistance; *OsWRKY26* was both up-regulated, which might mean both resistance and senescence. Both *OsWRKY69* and *OsWRKY93* showed up-resistance after infection but down-regulation during plant aging, which might mean resistance but no senescence (Table 1). Therefore, *OsWRKY69* and *OsWRKY93* were our favorite candidates for breeding of high yield and disease-resistant rice.

**TABLE 1:** Summary of the expression profiles of five *OsWRKYs* genes after pathogen infection and during plant aging.

**Genes**	**Expression profile response to *M. oryzae***	**Expression profile during aging**
*OsWRKY2*	Down	Down
*OsWRKY14*	Down	Up
*OsWRKY26*	Up	Up
*OsWRKY69*	Up	Down
*OsWRKY93*	Up	Down

### Evaluation of Disease Resistance of *OsWRKY93* Transgenic Lines to *Magnaporthe oryzae* Guy11

**FIGURE 4:**
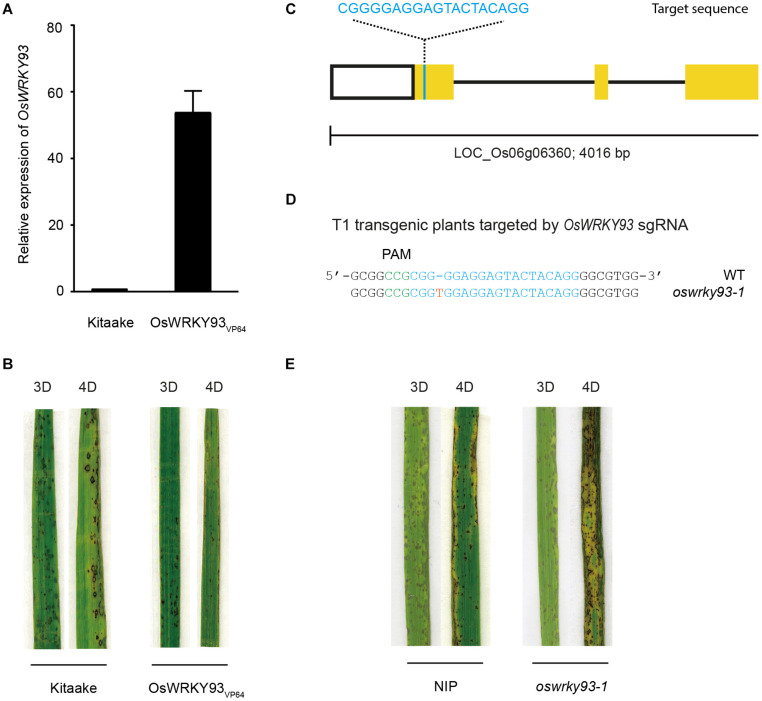
Generation and analysis of the *OsWRKY93* transgenic lines. **(A)** Real-time quantitative PCR experiments showing expression changes of *OsWRKY93* in Kitaake and the OsWRKY93_*V*__*P*__64_. **(B)** Representative leaves of Kitaake and the OsWRKY93_*V*__*P*__64_ 3 and 4 days after inoculation with *M. oryzae*. Pathogen infection assays were performed on three biological replicates. **(C)** Schematic diagram for the CRISPR-edited mutant of *OsWRKY93*. Yellow boxes and black lines represent exons and introns, respectively. The sgRNA target is cyan. **(D)** Sequence of the *oswrky93-1* mutant identified from transgenic plants of the *OsWRKY93* sgRNA target. The reverse complementary sequence of the PAM sequence (5’-CGG-3’) of the sgRNA target is green. The red T represents a one-base insertion. **(E)** Representative leaves of *Nipponbare* and *oswrky93-1* 3 and 4 days after inoculation with *M. oryzae*. Pathogen infection assays were performed on three biological replicates.

We showed that five *OsWRKYs* were induced in response to Guy11 treatment. In order to genetically evaluate five OsWRKYs protein functions, five *OsWRKY_*VP*__64_* transgenic lines were generated to explore the potential functions in rice disease resistance (see section “Materials and Methods”). We first detected their transcript levels of five *OsWRKY* genes by RT-qPCR. The results showed that five *OsWRKYs* genes all increased their transcript levels in the transgenic lines (*OsWRKYs _VP__64_*) compared with WT Kitaake (Figure 4A and Supplementary Figure S2). We then inoculated the three-leaf-stage *OsWRKYs_*VP*__64_* plants with *Magnaporthe oryzae* Guy11 using the spray-inoculation method. Surprisingly, we found that only *OsWRKY93_*V*__*P*__64_* plants showed a significant enhanced resistance to blast disease (Figure 4B). However, the other four of them have no significant alteration of disease resistance to *Magnaporthe oryzae* Guy11 in the transgenic lines (OsWRKYs_*VP*__64_) compared with WT Kitaake (Supplementary Figure S3).

In order to further confirm the role of *OsWRKY93* in disease resistance, we generated *oswrky93* mutants using CRISPR/Cas9 system in *Nipponbare* (Figure 4C). We found one mutant line *oswrky93-1* that carries a one-base insertion in the first exon of the *OsWRKY93* gene (Figure 4D). In contrast to *Nipponbare* plants, the CRISPR/Cas9-edited *oswrky93* mutants are more susceptible to *M. oryzae*, showing more disease lesions and less healthy leaf area (Figure 4E), suggesting that *oswrky93-1* plants exhibited elevated susceptibility to *M. oryzae*. Together with the results from the above analysis, these data imply the contribution of *OsWRKY93* to rice defense against *M. oryzae* infection.

### Detection of ROS Production in *OsWRKY93* Transgenic Lines

**FIGURE 5:**
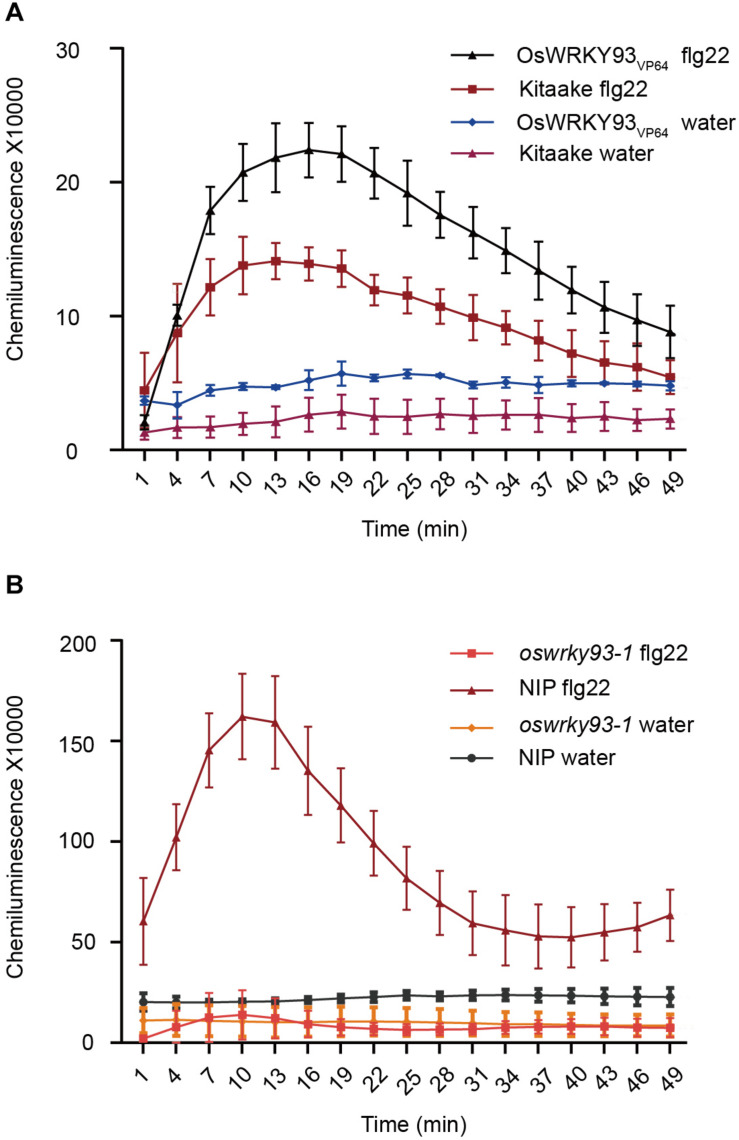
ROS accumulation in rice leaves after flg22 treatment. **(A)** A flg22-induced ROS burst in the OsWRKY93_*V*__*P*__64_ and Kitaake plants. **(B)** A flg22-induced ROS burst in the *oswrky93-1* and *Nipponbare* plants. Rice leaf disks were treated with 1 μM Flg22 or water. Error bars represents the SE (*n* = 3–5).

Reactive oxygen species (ROS) burst is a common feature in plant response to a number of biotic stresses, and flg22 has been shown to trigger ROS production in *Arabidopsis* (Mersmann et al., 2010). To examine whether enhanced-expression or knockout of *OsWRKY93* affect ROS production after flg22 treatment, we collected leaves from the *OsWRKY93_*V*__*P*__64_*, *oswrky93-1* and WT plants and measured immediately the ROS level after flg22 treatment. In our experiments, ROS production was increased in *OsWRKY93_*V*__*P*__64_* activation plants after treatment with flg22, and the flg22-induced ROS generation was twofold higher, compared to the Kitaake plants control and water treatment (Figure 5A). As expected, no constitutive ROS production was observed in *oswrky93-1* mutant plants (Figure 5B). Given these facts, we concluded that overexpressing *OsWRKY93* enhances PAMP-triggered immune response in rice.

### Detection of Darkness-Induced Leaf Senescence Phenotype in *OsWRKY93* Transgenic Lines

**FIGURE 6:**
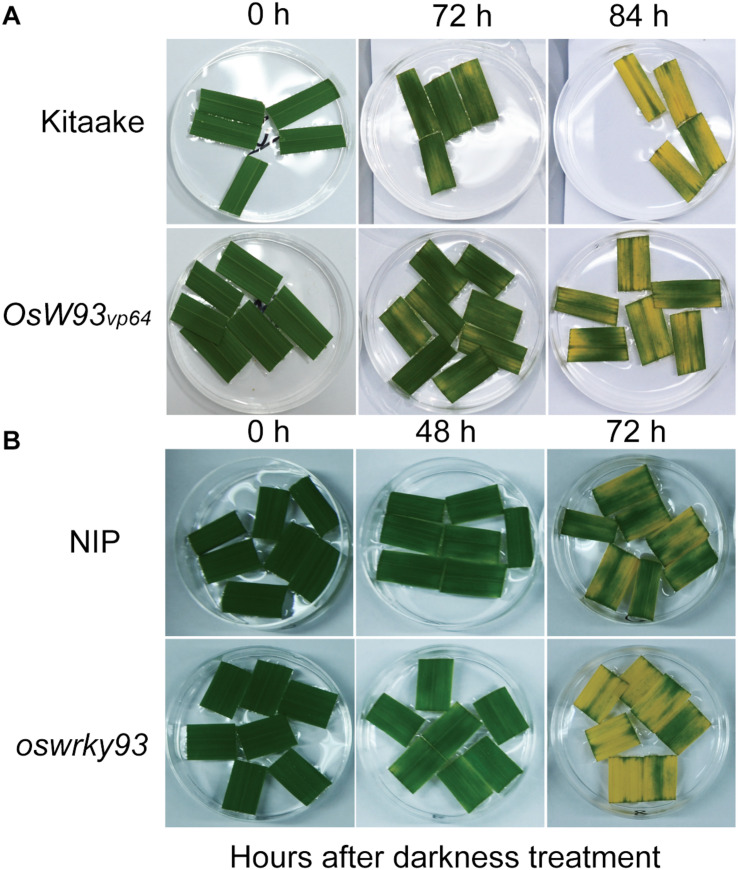
Phenotyping of detached leaves after darkness treatment. **(A)** A delaying leaf senescence shown in the OsWRKY93_*V*__*P*__64_ (*OsW93_*v*__*p*__64_*) compared to Kitaake plants. **(B)** An early leaf senescence shown in the *oswrky93-1* compared to *Nipponbare* (NIP) plants. Detached leaf pieces of rice were incubated with 1 μM MES (pH8.5) buffer after darkness treatment for 0, 48, 72, and 84 h.

In order to further evaluate the potential role of *OsWRKY93* in leaf senescence, the OsWRKY93_*v*__*p*__64_, *oswrky93-1* mutant and two ecotypes of rice (Kitaake and NIP) plants were used for phenotype observation. The plants grown in the soil during the period of 39 days after germination did not show any visibly different phenotypes among enhanced-expression or knockout of *OsWRKY93* and WT. However, the results of detached leaves after darkness treatment showed that the enhanced OsWRKY93 level clearly delayed leaf senescence after darkness treatment for 84 h in OsWRKY93_*v*__*p*__64_ line compared to Kitaake (Figure 6A), while knockout of *OsWRKY93* apparently promoted leaf senescence after darkness treatment for 72 h in the *oswrky93-1* line compared to NIP (Figure 6B). Therefore, *OsWRKY93* plays function in darkness induced leaf senescence, although there is no visible senescence phenotype in the seedling stage of *oswrky93* mutants.

In view of these facts, OsWRKY93 is a new candidate protein for in-depth understanding of the regulatory mechanisms of pathogen-induced cell death and leaf senescence, helping in breeding high-yield and disease-resistant crops.

## Discussion

Plant breeders are facing a serious challenge in rice production, that is, the premature senescence of leaves, in particular, flag leaves, which causes yield loss. There are, however, quite few studies that investigate the molecular mechanism of flag leaf senescence in rice. In this paper, we have identified 11 *OsWRKYs* that were differentially expressed during the senescence of flag leaves through RNA-Seq together with the RT-qPCR analysis. Importantly, we also surveyed the responses of 11 *OsWRKY* genes to *M. oryzae* to explore the correlation between leaf senescence and plant defense. Finally, we genetically identified OsWRKY93 as a new candidate protein for in-depth understanding of the regulatory mechanisms of pathogen-induced leaf senescence, helping in breeding high-yield and disease-resistant crops.

Our experimental results demonstrate that five senescence-inducible genes, *OsWRKY2*, *OsWRKY14*, *OsWRKY26*, *OsWRKY69*, and *OsWRKY93*, were induced in response to *M. oryzae* infection, implying that part of OsWRKY TFs connect leaf senescence and plant defense. In light of the fact that numerous studies have shown that the WRKY family plays a central role in leaf senescence as well as biotic stress tolerance (Bakshi and Oelmüller, 2014), it’s not surprising that some WRKY members might have dual functions between them, such as WRKY53, WRKY6, WRKY22, and WRKY70 in *Arabidopsis* (Robatzek and Somssich, 2002; Miao and Zentgraf, 2007; Rushton et al., 2010; Zhou et al., 2011; Hu et al., 2012; Chen J. et al., 2017; Zhou et al., 2018; Ramos et al., 2021). In this study, the transcript levels of *OsWRKY93* increased as leaf senescence progressed, suggesting that *OsWRKY93* is involved in the onset of flag leaf senescence. Gain-of *OsWRKY93* delays a dark-induced leaf senescence, contrary to the loss-of *OsWRKY93*, and promotes a dark-induced leaf senescence (Figure 6). We further showed that rice transgenic plants overexpressing *OsWRKY93* displayed an enhanced resistance to *M. oryzae* and the knockout *oswrky93-1* mutants are more susceptible to *M. oryzae*. In addition, we also found that the *OsWRKY93_*V*__*P*__64_* lines accumulated ROS highly in response to flg22 treatments (Figure 5A). In contrast, enhanced ROS production couldn’t be detected in the *oswrky93-1* mutant plants (Figure 5B). These results clearly indicate that the senescence-inducible gene *OsWRKY93* is also a positive regulator of the defense response in rice. These results also corroborate the findings of the previous study on *OsWRKY23*. As described in that paper, *OsWRKY23* was strongly induced by dark-induced senescence and its overexpression in Arabidopsis increased tolerance to pathogen infection (Jing et al., 2009). In addition, as we knew, plant senescence is controlled by genetically materials and influenced by environmental cues. In this study our RT-qPCR profiles of a few of 11 candidate WRKYs are not matched well with RNA-seq data (Figure 2), an uncontrollable growth condition of different years might be one of reasons for a few OsWRKY members sensitively in response to unknown environmental factors.

Phylogenetic analyses of the WRKY domain sequences provide support for the hypothesis that gene duplication of single- and two-domain WRKY genes and loss of the WRKY domain occurred in the evolutionary history of this gene family in rice (Xie et al., 2005). Based on the number of WRKY domains and the characteristics of the zinc-finger-like motif, the WRKY family can be divided into three types. According to amino acid sequence similarity, 97 WRKY proteins in *O. sativa* were divided into three types and 13 groups, of which class II WRKYs were divided into 10 subclasses (IIa–IIj), and class III WRKYs were divided into two subclasses (IIIa and IIIb) (Qiu et al., 2004; Rushton et al., 2010). It has been reported that class II or III WRKY members are mostly involved in plant defense response (Dong et al., 2003; Cheng et al., 2019; Wang et al., 2020). Here, OsWRKY2, OsWRKY14, and OsWRKY26 belonged to class II of the WRKY family. OsWRKY69 and OsWRKY93 belonged to class III of the WRKY family. Interestingly, we found that the expression profiles of five *OsWRKYs* genes were altered in both after pathogen infection and during plant aging, which showed that *OsWRKY2* was down-regulated: there was no resistance and no senescence; *OsWRKY14* was down-regulated after infection but up-regulated during plant aging: there was no resistance and senescence; *OsWRKY26* was up-regulated, with respect to both resistance and senescence; both *OsWRKY69* and *OsWRKY93* showed up-resistance after infection but were down-regulated during plant aging, with respect to resistance and no senescence (Table 1). Although the enhanced transgenic rice plants of *OsWRKY2*, *OsWRKY14*, and *OsWRKY26* did not show significantly changing phenotypes of infection to *M. oryzae* at seedling stage, it is possible they rely on a specific kind of pathogen or developmentally dependent. *OsWRKY69* and *OsWRKY93*, especially the latter, both are our favorite candidate genes for further in-depth understanding of their acting mechanism and the high yield and strong resistant genetically manipulation.

## Data Availability Statement

The raw data supporting the conclusions of this article will be made available by the authors, without undue reservation.

## Author Contributions

YL, SL, PM, YP, YZ, and XZ performed the research. YM and YL designed the research and analyzed the data. YM and YX wrote the manuscript. All authors contributed to the article and approved the submitted version.

## Conflict of Interest

The authors declare that the research was conducted in the absence of any commercial or financial relationships that could be construed as a potential conflict of interest.

## Supplementary Material

Supplementary Figure 1 Chlorophyll contents of rice flag leaves at six times during aging stages (0W, 1W, 2W, 3W, 4W, and 5W).

Supplementary Figure 2 The transcript levels of five enhanced expression OsWRKYs _*VP*__64_ transgenic lines compared to the Kitaake WT plants by RT-qPCR.

Supplementary Figure 3 The infection phenotypes of five enhanced expression OsWRKYs _*VP*__64_ transgenic lines to *M. oryzae.*

Supplementary Table 1 Primers used in this study.

Supplementary Table 2 Primers for genotyping CRISPR/Cas9 mutants.

Supplementary Dataset 1 The list of RPKM values and WRKY family DEGs.
