# Supplementary material for: OsWRKY93 Dually Functions Between Leaf Senescence and in Response to Biotic Stress in Rice
Source: Front Plant Sci. 2021 Mar 22;12:643011. doi: 10.3389/fpls.2021.643011 (PMC8019945; doi:10.3389/fpls.2021.643011)
Supplement: Supplementary Figure 1 — Chlorophyll contents of rice flag leaves at six times during aging stages (0W, 1W, 2W, 3W, 4W, and 5W). [file Data_Sheet_1.PDF]

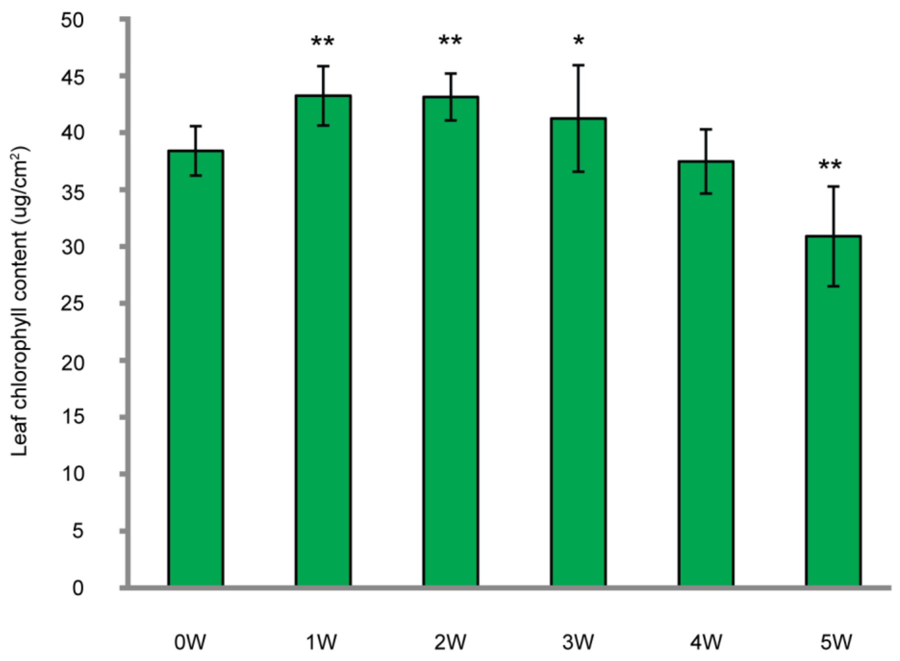

Supplementary Figure S1. Chlorophyll contents of rice flag leaves at six times during aging stages (0W, 1W, 2W, 3W, 4W and 5W). The error bars indicate standard deviation of three biological replicates and five technique replicates were used. Asterisk indicate significant differences (*P < 0.05 and **P<0.01) based on Student's t-test compared to WT.

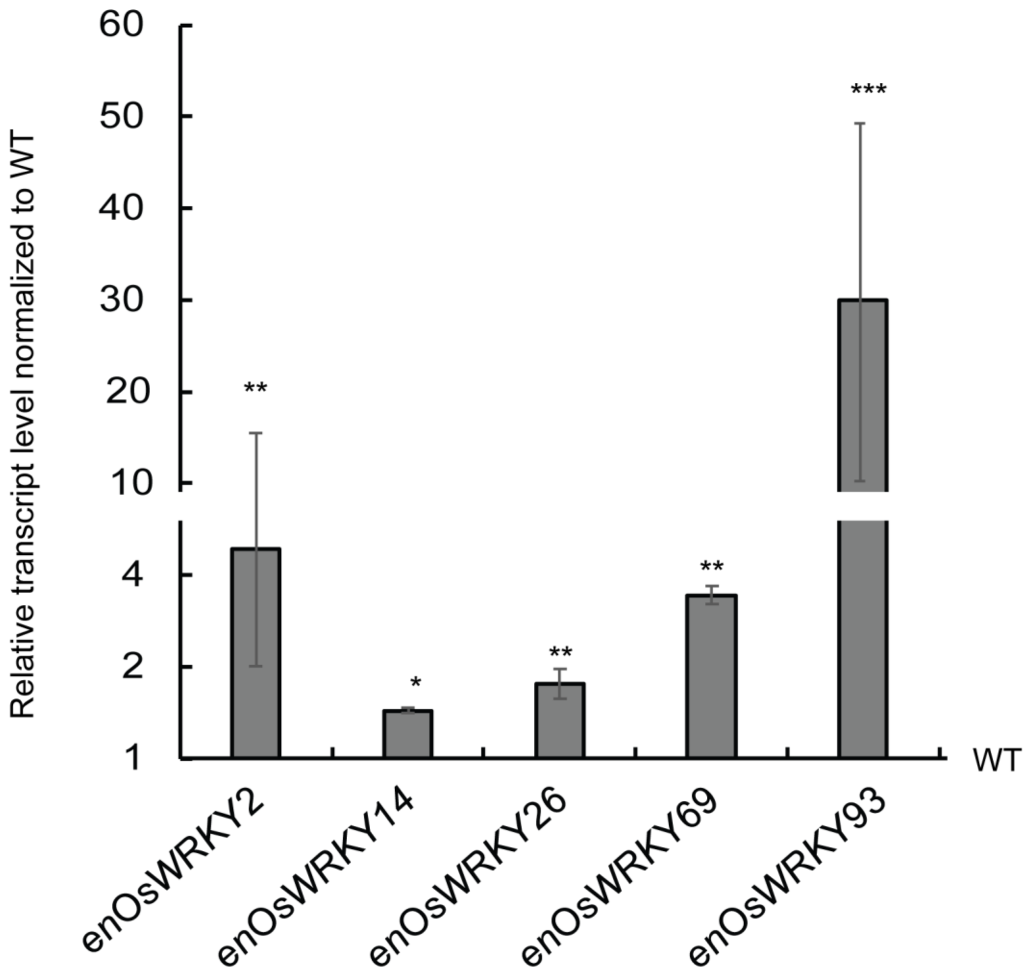

Supplementary Figure S2. The transcript levels of five enhanced expression OsWRKYs VP64 transgenic lines compared to the Kitaake WT plants by RT-qPCR The error bars indicate standard deviation of three biological replicates and three technique replicates were used. Asterisk indicate significant differences (*P < 0.05 and **P<0.01) based on Student’s t-test compared to WT

.

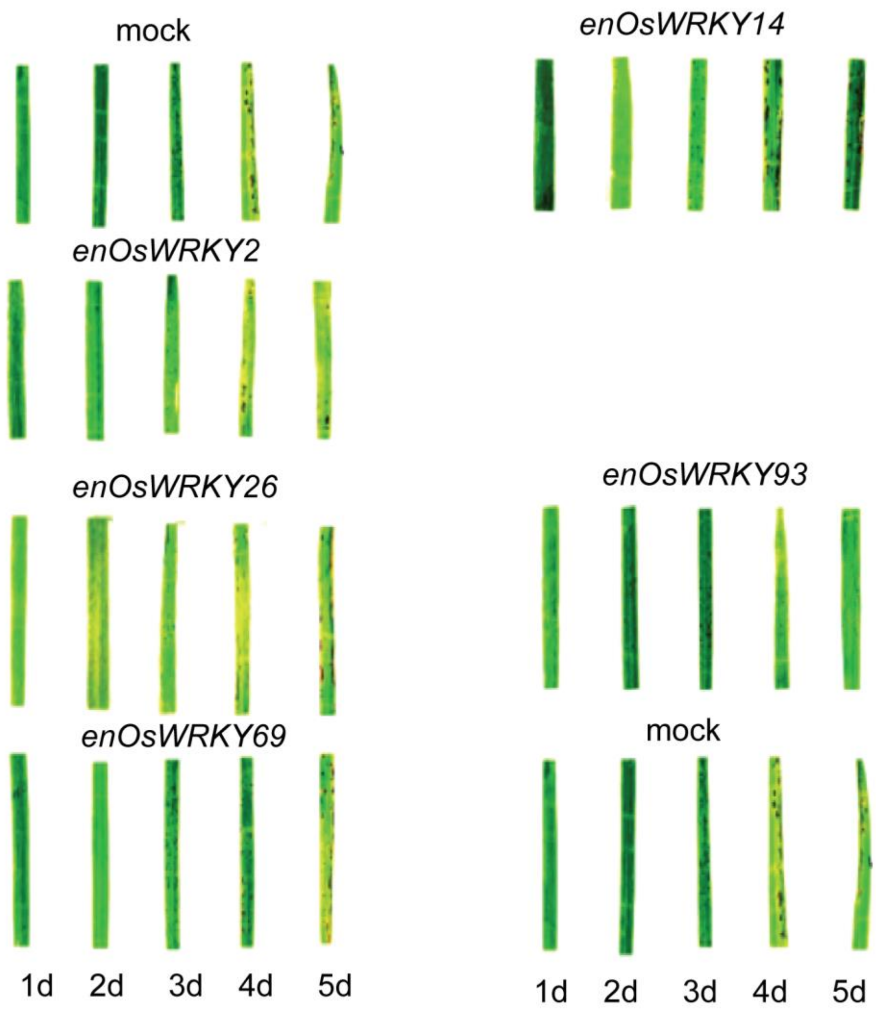

Supplementary Figure S3. The infection phenotypes of five enhanced expression *OsWRKYs* $_{VP64}$ transgenic lines to *M. oryzae*

Representative leaves of Kitaake and the *OsWRKYvp6*4 after inoculation with *M. oryzae* for 1, 2, 3, 4, 5 days. Pathogen infection assays were performed on three biological replicates.

Table S1. The list of primer pairs used in this study.

Gene Names	Locus Names	Sequences
OsWRKY2	LOC_Os10g42850	F: CTACACCTCCGACCACAACCA
		R: GTAAGACGATCGTGGCCAGTCT
OsWRKY10	LOC_Os01g09100	F:CGCACTCGTCGGAATCGT
		R:AGTGCATACAAGAACCGATCGA
OsWRKY14	LOC_Os01g53040	F:CACCTACTCCTTCGAGCACA
		R:GTTTCCTCGGGTTCCACTT
OsWRKY26	LOC_Os01g51690	F:GTGGAGGAAGTACGGGAAAAAGT
		R:TGGATCCCCTCGTACATCGT
OsWRKY29	LOC_Os07g02060	F:GCCAGAAAGCCGTCAAGAAC
		R:CGGTGTGGTGGGAGTGTTG
OsWRKY45	LOC_Os05g25770	F: CGGGTAAAACGATCGAAAGA
		R: TTTCGAAAGCGGAAGAACAG
OsWRKY47	LOC_Os07g48260	F:AGCGCCTGCGGATTACG
		TAAGGATCGAAGCCAAACATGTC
OsWRKY49	LOC_Os05g49100	F:GGTTCGCGTTCATGACCAA
		R:CGCTCCACCCTCTTCTTCAC
OsWRKY69	LOC_Os08g29660	F:GTAAGATCAGTGCAGGTGTAAGCAGTA
		R:CTTCTTATTTTCCTCTCTTTTGCTTGA
OsWRKY72	LOC_Os11g29870	F:TGCATGGATCGATGGATGTTA
		R:GAATCTTGCTTGCCACTGAATCT
OsWRKY73	LOC_Os06g05380	F:TGATGACGTTGAGCCCTGAA
		R:TGACCTGTGGCTGAGGAGCTA
OsWRKY93	LOC_Os06g06360	F:AACGCCACCATGACACGAT
		R:GGAATTTGGCACCGAGGAT
OsNAC4	LOC_Os07g04560	F:TCCTGCCACCATTCTGAGATG
		R:TTGCAGAATCATGCTTGCCAG
OsACTIN	LOC_Os03g50885	F:CCAAGGCCAATCGTGAGAAGA

		R:AATCAGTGAGATCACGCCCAG

Table S2. The list of primer pairs for genotyping CRISPR/Cas9 mutants.

Gene Names	Locus Names	Sequences
OsWRKY93	LOC_Os06g06360	F:CTTAGCTTTCGCGTGTCGTG
		R:GAACACACACACGCACCTTC
